# Fulminant group A streptococcal necrotizing fasciitis presenting as severe calf pain after pharyngitis in an adolescent: a case report

**DOI:** 10.3389/fmed.2026.1906805

**Published:** 2026-09-03

**Authors:** Lisa Roncoroni, Jan Kühle, Lampros Kousoulas, Katharina Müller-Peltzer, Dawid L. Staudacher

**Affiliations:** Interdisciplinary Medical Intensive Care (IMIT), University of Freiburg Medical Center, Freiburg, Germany

**Keywords:** case report, Group A Streptococcus, interdisciplinary approach, necrotizing fasciitis (NF), streptococcus toxic shock syndrome, calf pain

## Abstract

**Background:**

Invasive Group A Streptococcus (GAS) infection can rapidly progress to life-threatening conditions, including necrotizing fasciitis (NF) and streptococcal toxic shock syndrome (STSS). While typically associated with cutaneous breaches or underlying comorbidities, hematogenous seeding following primary pharyngeal infection is a rare but documented phenomenon. Early diagnosis is often complicated by subtle skin changes and the rarity of the disease.

**Case:**

A previously healthy 17-year-old female presented with acute pain of the right calf accompanied by severe tachycardia and rapidly progressive hypotension. Notably, she had experienced acute pharyngitis symptoms approximately two days prior to the onset of her soft tissue pain. Physical examination revealed no typical cutaneous hallmarks of necrotizing fasciitis, such as severe erythema, skin discoloration or crepitus. Complicating the diagnosis, radiological imaging strongly suggested acute cholecystitis as an alternative potential source of her systemic sepsis. Due to the absence of conventional typical signs and the presence of a confounding abdominal focus, establishing a diagnosis of NF proved to be an exceptional clinical challenge; the correct diagnosis was ultimately made possible only thanks to a rapid, multidisciplinary discussion involving emergency medicine, radiology and surgical teams. Emergency surgical exploration of the right lower extremity confirmed widespread necrosis of the superficial fascia and subcutaneous tissue. Cultures from both the deep fascial tissue and blood grew monomicrobial *Streptococcus pyogenes*. The absence of any local skin trauma supported a diagnosis of hematogenous seeding from the recent pharyngeal infection. The patient was aggressively managed in the intensive care unit with multiple rounds of emergency surgical debridement, targeted intravenous antibiotic therapy (high-dose penicillin and clindamycin), vasoactive support, and adjuvant intravenous immunoglobulin (IVIG) therapy. Following a prolonged hospital course, the patient stabilized, recovered completely, and was successfully discharged.

**Conclusion:**

Severe pain disproportionate to skin findings after a recent streptococcal infection should prompt consideration of necrotizing fasciitis, even in otherwise healthy adolescents. Early surgical exploration and source control remain central to management, while adjunctive IVIG may be considered in selected patients with suspected streptococcal toxic shock syndrome.

## Introduction

Necrotizing fasciitis (NF) is a challenging clinical infection. It is characterized by the rapid spread of extensive necrosis of the skin and underlying structures. The infection typically propagates along the muscle fascia due to its relatively poor blood supply.

NF can be classified into two categories based on microbiology: polymicrobial (NF type I) and monomicrobial (NF type II). NF type II is typically caused by Group A Streptococcus (GAS) or other beta-hemolytic streptococci. The incidence of invasive GAS disease varies between European countries, ranging from 1.5 and 3.5 cases per 100,000 persons (1). Superficial infections constitute the predominant category of pediatric cases of GAS disease, exhibiting a predominantly benign and self-limiting nature. Rare is the case of an invasive infection, which is often complicated by multi-organ failure and shock, and is associated with high mortality (2).

Managing patients with NF is a challenge for caregivers. First, because the infection originates in the deep subcutaneous tissue, superficial skin findings may appear deceptively mild, leading to underdiagnosis. Second, despite appropriate antibiotic therapy, the prognosis remains poor without prompt surgical debridement. Third, due to the rarity of NF clinicians may not consider it as an early differential diagnosis. Fourth, Imaging may support diagnosis, but clinical suspicion and surgical exploration remain decisive.

This case is reported to emphasize that invasive GAS necrotizing fasciitis may occur in an otherwise healthy adolescent after apparently self-limited pharyngitis and may initially present with severe limb pain and minimal cutaneous abnormalities.

## Presentation of case

A 17-year-old female presented to the emergency department with severe pain in the right calf, resulting in a limited ability to walk. The patient had been afebrile for 1–2 days and become increasingly weak. A few days earlier, the patient experienced upper respiratory symptoms, including a sore throat and fever. The patient's brother had also been ill, but had recovered completely within a few days. There was no record of significant medical history. The patient denied any antecedent trauma, insect bite, burns or long-term medication.

On arrival, the patient's vital signs were as follows: temperature 36,1°C, heart rate 140/min, blood pressure 91/48 mmHg, respiratory rate 40/min, oxygen saturation 97%. She was agitated and panicked. Examination of the right calf showed severe tenderness. Dorsal extension was impossible due to severe pain. The calf was swollen and firm with increased circumference; no erythema, bullae, crepitus, skin necrosis or wound was present.

Bedside ultrasound did not demonstrate findings diagnostic of deep vein thrombosis (DVT), although the examination was limited because compression of the calf veins was not tolerated. Echocardiography showed moderately impaired left ventricular pumping function with global hypokinesis. There was no evidence of right ventricular strain, making high-risk pulmonary embolism (PE) less likely. Due to the young age of the patient and the lack of evidence of hemodynamically relevant PE, we initially decided against a CT scan.

The patient rapidly developed a vasoplegic shock (mean arterial pressure [MAP] below 45 mmHg). Volume resuscitation (initial bolus of 20 ml/kg body weight using a pressure bag; total replacement volume of approximately 2 L) and noradrenalin-support up to 0.14 µg/kg/min was initiated. After her condition stabilized (MAP around 70 mmHg), the patient was transferred to our intensive care unit.

**Figure 1:**
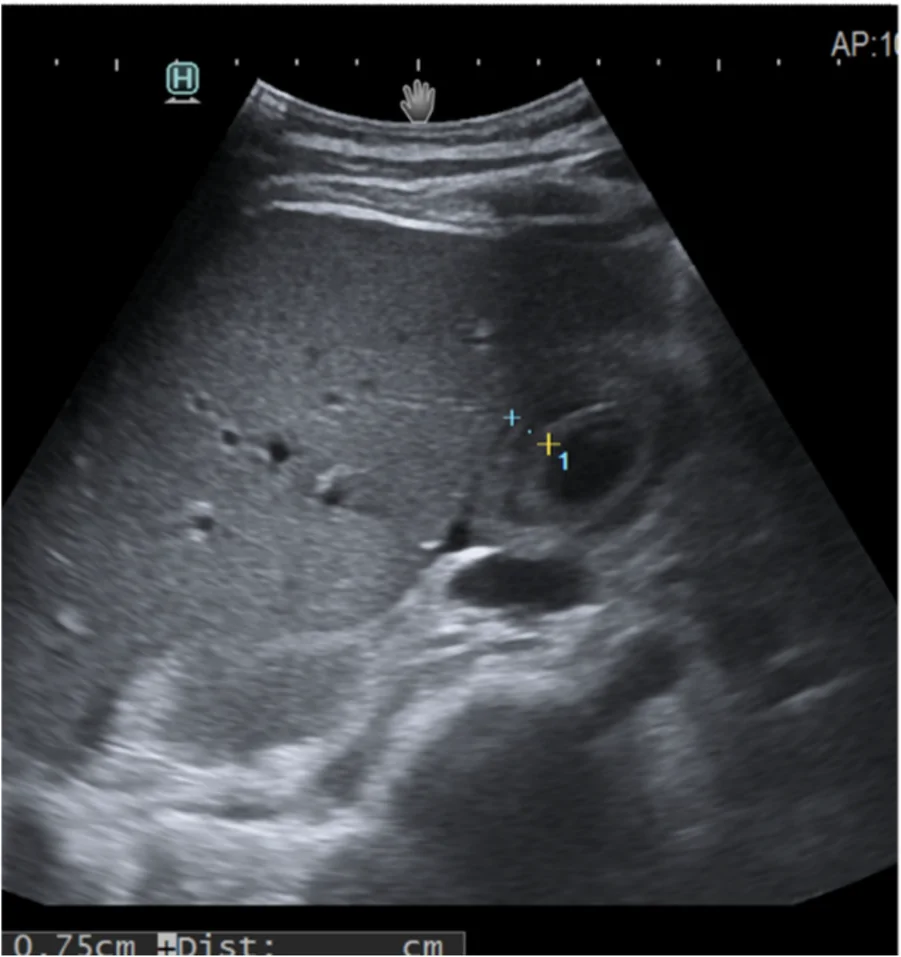
Ultrasound findings suggestive of cholecystitis.

Blood tests on admission are reported in Table 1. Laboratory and clinical tests suggested a sepsis with multiple organ failure. Following the collection of microbiological samples, empirical antibiotic therapy was initiated with piperacillin/tazobactam (4.5 g i.v. thrice daily) due to suspected septic shock. Within the first 12 h after admission to hospital, the patient's condition deteriorated, requiring additional vasopressor support with vasopressin (max. 10 µg/h). Despite broad antibiotic therapy, the severe septic shock worsened, suggesting an unaddressed focus. Visually, the right leg appeared normal despite the persistent swelling and a slightly regressive pain. Abdominal ultrasound findings were highly suggestive of cholecystitis (see Figure 1). However, acute cholecystitis was questioned clinically due to the lack of peritonism, and the sonographic abnormalities could have been caused by shock-related interstitial oedema of the bile ducts and gallbladder.

**Table 1:** Blood test results on admission and LRINEC score.

Parameters	Blood test results	LRINEC- score		Points
White blood cells (WBC)	20,040/µL (↑)	<15,000/µl	0	+1
		15,000–25,000/µL	+1	
		≥25,000/µL	+2	
Hemoglobin	11.6 g/dl (↓)	>13.5 g/dl	0	+1
		11–13.5 g/dl	+1	
		<11 g/dl	+2	
C-reactive protein	173 mg/dl (↑)	<150 mg/dl	0	+4
		≥150 mg/dl	+4	
Procalcitonin	11.2 ng/ml (↑)			
D-Dimer	2.7 µg/ml (↑)			
Creatinine	2.05 mg/dl (↑)	≤1,6 mg/dl	0	+2
		˃1,6 mg/dl	+2	
Sodium	133 mmol/l (↓)	≥135 mmol/l	0	+2
		<135 mmol/l	+2	
Glucose	85 mg/dl	≤180 mg/dl	0	0
		˃180 mg/dl	+1	
Aspartate transaminase (AST)	46 IU/L (↑)			
Alanine transaminase (ALT)	49 IU/L (↑)			
Gamma-GT	190 U/L (↑)			
Alkaline phosphatase (ALP)	185 IU/L (↑)			
Bilirubin	3.3 mg/dl (↑)			
Total				10

**Figure 2:**
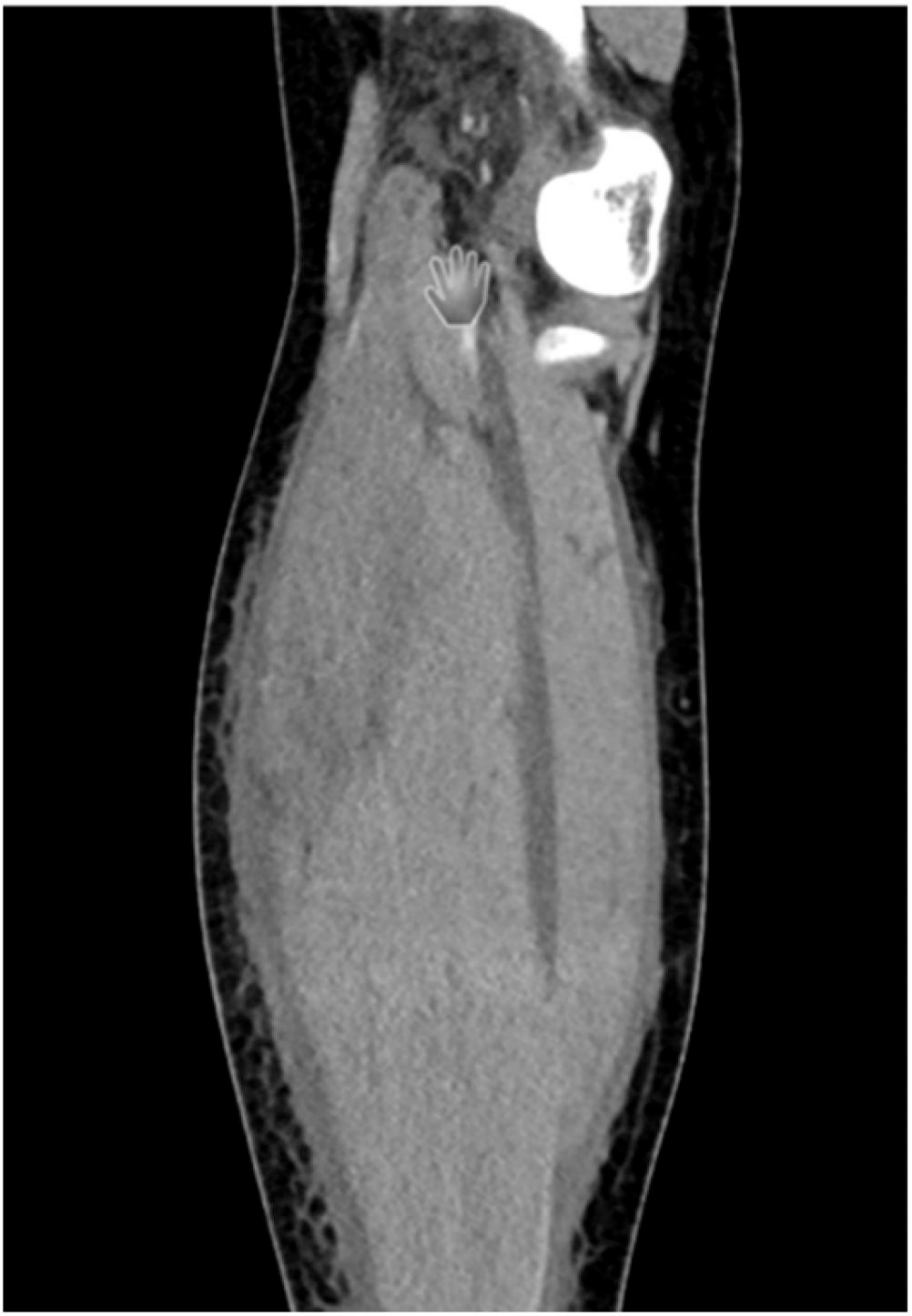
Coronal CT reconstruction in venous phase of the right calf, demonstrating perifascial fluid and hypodense areas within the right gastrocnemius muscle.

For further assessment, CT imaging was performed and confirmed periportal edema with pronounced thickening and contrast enhancement of the gallbladder wall. Additionally, perifascial fluid and hypodensity in the right gastrocnemius indicated a diffuse inflammatory process, raising suspicion for necrotizing fasciitis (see Figure 2). Pulmonary embolism was excluded.

Following an interdisciplinary bedside team decision involving specialists in intensive care, radiology, orthopedics and abdominal surgery, it was deemed necessary to perform emergency surgery. Both potential foci — cholecystitis and NF — were considered plausible and warranted surgical intervention. These were managed in a single interdisciplinary emergency surgery approximately 15 h after admission.

A cholecystectomy was performed without complications; the gallbladder showed signs of oedema and inflammation, with some fibrin deposits, which were consistent with acalculous cholecystitis.

Extensive debridement of the right lateral anterior calf was performed until healthy, bleeding tissue was reached down to the muscle. Initially, a 10 cm incision was made in the lower outer leg. The subcutaneous fascia was visibly thickened in patches, indicating a reduced blood supply. Therefore, the incision was extended to cover the entire lower leg. Upon opening the fascia, cloudy secretion was seen to drain out. Samples were taken from the fascia of the underlying necrotic muscle in the lateral gastrocnemius region, as well as from the surrounding subcutaneous tissue. Blunt dissection was then performed along the fasciae, opening the deep dorsal fascial compartment, followed by the medial and anterior fascial compartments. All the fascia appeared thickened, inflamed and distended and showed no tendency to bleed when incised. Extensive debridement of the fascia was performed, with all necrotic sections removed. Extensive resection was performed on the muscle within the necrotic areas. Once healthy tissue was exposed, the area was extensively irrigated. This was followed by the application of a VAC sponge and dressing.

Following surgery, septic shock persisted, as evidenced by hypotension necessitating catecholamine support, respiratory compromise requiring invasive ventilation (initially BiPAP-ASB with FiO2 max. 40% and PEEP 10), and capillary leakage necessitating fluid resuscitation and resulting in a positive fluid balance. Furosemide was administered intermittently to achieve a consistent negative fluid balance. Following an initial brief period of anuria, spontaneous diuresis resumed, meaning that renal replacement therapy was not required.

Twenty-two hours after admission, blood cultures identified Streptococcus pyogenes as the causative agent of NF. The microbiological examination of the leg biopsy also revealed the presence of Streptococcus pyogenes in almost all samples. However, the throat swab remained negative.

Antibiotic therapy was adjusted to high-dose penicillin G (5 million units intravenously four times a day) combined with clindamycin (900 mg intravenously three times a day).

Intravenous immunoglobulins were administered for streptococcal toxic shock syndrome (STSS) at 1 g/kg on day 1 and 0.5 g/kg on days 2 and 3.

The anti-infective treatment described above resulted in a rapid improvement in the patient's general condition. Daily surgical debridement and VAC system changes were performed until day 5.

On day 5, the patient was extubated. She remained stable thereafter whilst receiving supplemental oxygen at a rate of 1–2 L/min via a nasal cannula. She was given analgesic treatment with continuous hydromorphone, which was subsequently tapered off in favor of metamizole and oxycodone/naloxone.

On day 11, the patient was discharged to the paediatric ward in an improved overall state of health.

Intravenous antibiotic therapy was continued cumulatively for three weeks.

A number of additional surgical wound debridements were performed, interspersed with dermal traction to minimize the wound area during the treatment process. Intraoperative findings indicated that direct split-skin grafting was not feasible at that stage. Instead, during a further surgical revision on day 31, a dermal substitute (NovoSorb®) was inserted, and VAC therapy was continued to promote healing. On day 37, the VAC dressing was replaced with a fat-gauze dressing.

The patient was then discharged on day 38 and further orthopedic therapy continued on an outpatient basis.

At discharge, the patient was mobile using forearm crutches while still wearing a cast on her right foot, which was kept in flexion to prevent equinus deformity. Partial weight-bearing of up to 25 kg was permitted. The patient was prescribed continuous physiotherapy, including calf muscle strengthening and gait training.

Twenty-five days after discharge the patient was readmitted for elective surgery to cover the defect on the right lower leg with a split-thickness skin graft, harvested from the lateral thigh down to the knee. VAC therapy was administered again for six days to improve wound healing. Once the VAC pump had been removed, the patient was discharged home and transitioned to full weight-bearing, only using the positioning splint at night.

At the two-month follow-up appointment, the patient was able to walk independently, with no neurological deficit or recurrent infection. Complete epithelisation of the split-thickness skin graft had occurred, and no additional debridement or readmission was required.

## Discussion

GAS infections became more prevalent in industrialized nations during the 1980s and 1990s, earning the bacteria the colloquial term “flesh-eating” (2). The most at-risk groups for invasive disease are the elderly population and young children, especially those under one year of age (3, 4). A broad range of illnesses of differing degrees of severity is the result of GAS infection, from minor skin infections and pharyngitis to life-threatening invasive diseases.

A patient with NF typically presents with three main symptoms in the area of the entry point: pain, swelling, and erythema (5).

The severity of the pain is typically disproportionately high in relation to the physical manifestations observed, and patients may also experience sensory impairment in the affected area. In the early stages of the infection (i.e., 1–2 days afterwards), bullae and vesicles may appear, accompanied by serosanguinous leakage (the intermediate stage of NF). This is followed by skin necrosis and crepitus (the late stage) (6, 7).

A recent retrospective study (8) of 69 adult patients revealed that 84% of the patients experienced pain, 75% exhibited erythema and 72% displayed edema and swelling. Crepitus on examination was less common and observed in fewer than 50% of the patients. An early diagnosis was enabled by these common clinical findings. Overall mortality was 26.5%, significantly influenced by predisposing factors such as diabetes mellitus, hemodynamic instability at admission, and polymicrobial infections.

Our patient most likely had ordinary streptococcal pharyngitis at onset, followed by hematogenous spread and the development of STSS. The negative throat swab result was likely a false negative. Physical examination revealed swelling and severe pain in her lower leg, but there were no signs of redness, erythema, or pre-existing skin lesions. There were no additional risk factors such as intravenous drug use, immunosuppression or diabetes.

Although critical progression of NF in immunocompetent hosts has been documented, as recently reported by Vasudevan et al. (9), our case is distinguished by the almost complete absence of dermatological clinical findings.

Based on the clinical findings, we initially suspected a DVT with possible PE. However, due to the patient's young age and the absence of evidence of a hemodynamically significant PE, we initially opted against a CT scan.

It quickly became apparent, however, that an infection was the underlying cause; therefore, we started empirical broad-spectrum antibiotic therapy within two hours of the patient's admission to the hospital. Laboratory and clinical tests indicated sepsis with multiple organ failure, including septic cardiomyopathy, oliguric renal failure, and liver failure with coagulopathy.

In view of the suggestive ultrasound findings of cholecystitis, an abdominal focus was suspected. However, the absence of peritonism during the physical examination led us to question the possible diagnosis. Clinically we could not find an alternative focus. Despite the swelling, the right leg appeared normal with no sign of infection. The pain in the limb also slightly improved.

Thanks to an interdisciplinary case discussion, NF was pointed out as tentative diagnosis soon and after radiological imaging an emergency surgical intervention was scheduled.

Due to the atypical clinical presentation (absence of erythema, bullae, crepitus, skin necrosis or wound) in conjunction with the potential alternative infection source, as indicated by the highly suggestive radiological imaging, we elected to perform a cholecystectomy and exploratory limb surgery in a single procedure. NF could only be confirmed after surgical examination.

Despite the lack of histopathological findings, it can be speculated that the cholecystitis was, in all likelihood, a reactive phenomenon and that the cholecystectomy was unnecessary.

In the setting of STSS, multi-organ dysfunction and systemic hypoperfusion can frequently lead to reactive, acalculous cholecystitis or gallbladder wall thickening due to generalized capillary leak syndrome. In this case, the gallbladder findings acted as a major confounding factor.

In retrospect, it became clear that the findings of the clinical examination (i.e., disproportionate pain) and the patient's medical history (i.e., fever and sore throat) were underestimated initially. However, bacteremia as a result of streptococcal pharyngitis is only observed in 0.3% of pediatric patients with fever (7).

Currently, there are no published guidelines for optimally treating NF. Many biomarkers and scoring-systems have been evaluated, with the Laboratory Risk Indicator for Necrotizing Fasciitis (LRINEC) being a notable example, as it incorporates serum white blood cell count, hemoglobin, sodium, glucose, creatinine, and C-reactive protein (CRP). A score of 6 or higher is strongly associated with NF, with a positive predictive value of 96% (10). In our patient the LRINEC score was 10 (s. Table 1). However, the validity of this scoring tool has been called into question by larger studies, which have demonstrated limited sensitivity (11–13). Although laboratory tests and imaging tools can assist in diagnosing NF, it is essential to recognize that they should be regarded as additional to clinical assessment. The gold standard method for confirming NF is surgical exploration, which reveals specific surgical findings, such as “dull gray necrotic tissue and fascia, pus resembling dishwater, non-contracting muscle, absence of bleeding, or a positive finger test, whereby that tissue can be easily separated with a gloved finger” (5).

It is noteworthy that GAS continues to demonstrate sensitivity to penicillin, despite the extensive utilization of this antibiotic (14). In the management of invasive GAS disease, the addition of clindamycin to penicillin therapy has been demonstrated to be advantageous. There exists a dual mechanistic rationale for this phenomenon. Firstly, while GAS exhibits exquisite sensitivity to penicillin *in vitro*, its *in vivo* efficacy is constrained by the “inoculum effect”. The latter is a term used to describe the phenomenon where the effectiveness of an antibiotic is reduced as the density of bacteria increases (15). Conversely, clindamycin exhibits no discernible sensitivity to variations in inoculum size. Secondly, clindamycin inhibits bacterial protein synthesis, which plays a significant role in the pathogenesis of GAS, especially in the case of STSS (14–16).

Although there is currently limited evidence to support the use of intravenous immunoglobulin (IVIG) and hyperbaric therapy in these settings, these treatments may still be beneficial. IVIG has been proposed as an adjunctive therapy in cases of STSS to reduce the high mortality rate characteristic of this condition. The bactericidal activity of serum has been shown to be enhanced by IVIG, which facilitates bacterial opsonization, neutralizes superantigens and toxins, stimulates leukocytes, and exerts a general anti-inflammatory effect through its effects on Fc receptor expression, complement, cytokines, and B and T cells (17).

Because STSS was suspected, IVIG was administered as adjunctive therapy. Given the limited and heterogeneous evidence supporting IVIG in STSS, the favorable outcome in this case should not be attributed to IVIG alone. Early surgical source control, appropriate antimicrobial therapy, and intensive care support were likely central to recovery.

In view of the severity of illness that can result from STSS and the fairly good safety profile of IVIG, along with the proof suggesting possible benefit of IVIG, it should nevertheless be regarded as an additional therapy in cases of STSS.

The findings of this case report must be interpreted in light of several limitations. First, the evidence is based on a single patient—a previously healthy teenager with no comorbidities—which precludes extrapolating these outcomes to a broader population suffering from NF with STSS. Second, we were unable to definitively confirm a primary streptococcal pharyngitis as the inciting event due to the negative throat swab result. We relied on the patient's recollection of the preceding symptoms of a sore throat and fever. Additionally, given the descriptive nature of this study and the simultaneous administration of multimodal therapies, including emergency surgical debridement and broad-spectrum antibiotics, it is impossible to infer a definitive causal benefit from the use of intravenous IVIG alone. The limited generalizability of this case is further compounded by the patient's atypical clinical presentation and rapid progression. These factors may be indicative of unique host-pathogen interactions or highly virulent strains of Streptococcus pyogenes that differ from the standard clinical course.

## Conclusion

This case highlights that GAS necrotizing fasciitis with STSS may occur in an otherwise healthy adolescent after apparent pharyngitis and may initially present with severe limb pain despite minimal skin findings. When pain is disproportionate to examination findings and systemic deterioration develops, clinicians should maintain suspicion for NF and pursue early surgical consultation. Adjunctive IVIG may be considered in selected cases of suspected STSS, but broader conclusions require stronger evidence.

## Data Availability

The original contributions presented in the study are included in the article/Supplementary Material, further inquiries can be directed to the corresponding author/s.

## References

[1] Nawijn F De Gier B Brandwagt DAH Groenwold RHH Keizer J Hietbrink F. Incidence and mortality of necrotizing fasciitis in The Netherlands: the impact of group A Streptococcus. BMC Infect Dis. (2021) 21:1217. 10.1186/s12879-021-06928-5 34872527 PMC8650531

[2] Carapetis JR Steer AC Mulholland EK Weber M. The global burden of group a streptococcal diseases. Lancet Infect Dis. (2005) 5(11):685–94. 10.1016/S1473-3099(05)70267-X 16253886

[3] O'Loughlin RE Roberson A Cieslak PR Lynfield R Gershman K Craig A. The epidemiology of invasive group a streptococcal infection and potential vaccine implications: united States, 2000–2004. Clin Infect Dis. (2007) 45(7):853–62. 10.1086/521264 17806049

[4] O’Grady K-AF Kelpie L Andrews RM Curtis N Nolan TM Selvaraj G. The epidemiology of invasive group a streptococcal disease in Victoria. Aust Med J Aust. (2007) 186:565–9. 10.5694/j.1326-5377.2007.tb01054.x 17547544

[5] Hua C Urbina T Bosc R Parks T Sriskandan S De Prost N. Necrotising soft-tissue infections. Lancet Infect Dis. (2023) 23:e81–94. 10.1016/S1473-3099(22)00583-7 36252579

[6] Wong CH Wang YS. The diagnosis of necrotizing fasciitis. Curr Opin Infect Dis. (2005) 18:101–6. 10.1097/01.qco.0000160896.74492.ea 15735411

[7] Stevens DL Bryant AE Goldstein EJ. Necrotizing soft tissue infections. Infect Dis Clin North Am. (2021) 35:135–55. 10.1016/j.idc.2020.10.004 33303335

[8] Celik K Danis F Kudu E. Predicting mortality in necrotizing fasciitis: retrospective evaluation of 69 cases. North Clin Istanb. (2025) 12(3):307–13. 10.14744/nci.2024.94557 40843331 PMC12365481

[9] Vasudevan B Das P Das NK Bhatnagar A Mukhida S Paliwal D. A case of necrotizing fasciitis with disseminated intravascular coagulation in an immunocompetent adult. J Dermatol Nurs Assoc. (2025) 17(4):121–6. 10.1097/JDN.0000000000000840

[10] Wong C-H Khin L-W Heng K-S Tan K-C Low C-O. The LRINEC (laboratory risk indicator for necrotizing fasciitis) score: a tool for distinguishing necrotizing fasciitis from other soft tissue infections. Crit Care Med. (2004) 32:1535–41. 10.1097/01.CCM.0000129486.35458.7D 15241098

[11] Hsiao C-T Chang C-P Huang T-Y Chen Y-C Fann W-C. Prospective validation of the laboratory risk indicator for necrotizing fasciitis (LRINEC) score for necrotizing fasciitis of the extremities. PLoS One. (2020) 15:e0227748. 10.1371/journal.pone.0227748 31978094 PMC6980593

[12] Fernando SM Tran A Cheng W Rochwerg B Kyeremanteng K Seely AJE. Necrotizing soft tissue infection: diagnostic accuracy of physical examination, imaging, and LRINEC score: a systematic review and meta-analysis. Ann Surg. (2019) 269:58–65. 10.1097/SLA.0000000000002774 29672405

[13] Bechar J Sepehripour S Hardwicke J Filobbos G. Laboratory risk indicator for necrotising fasciitis (LRINEC) score for the assessment of early necrotising fasciitis: a systematic review of the literature. Ann R Coll Surg Engl. (2017) 99:341–6. 10.1308/rcsann.2017.0053 28462647 PMC5449710

[14] Allen U Moore D. Invasive group a streptococcal disease: management and chemoprophylaxis. Paediatr Child Health. (2010) 15(5):295–302. 10.1093/pch/15.5.295 21532795 PMC2912623

[15] Goscinski G Tano E Thulin P Norrby-Teglund A Sjolin J. Release of SpeA from Streptococcus pyogenes after exposure to penicillin: dependency on dose and inhibition by clindamycin. Scand J Infect Dis. (2006) 38(11e12):983–7. 10.1080/00365540600836997 17148065

[16] Mascini EM Jansze M Schouls LM Verhoef J Van Dijk H. Penicillin and clindamycin differentially inhibit the production of pyrogenic exotoxins A and B by group A streptococci. Int J Antimicrob Agents. (2001) 18(4):395–8. 10.1016/S0924-8579(01)00413-7 11691576

[17] Takei S Arora YK Walker SM. Intravenous immunoglobulin contains specific antibodies inhibitory to activation of T cells by staphylococcal toxin superantigens. J Clin Invest. (1993) 91(2):602–7. 10.1172/JCI116240 8432865 PMC287991

